# Application of hyperspectral imaging for spatial prediction of soluble solid content in sweet potato

**DOI:** 10.1039/c9ra10630h

**Published:** 2020-09-08

**Authors:** Yuanyuan Shao, Yi Liu, Guantao Xuan, Yongxian Wang, Zongmei Gao, Zhichao Hu, Xiang Han, Chong Gao, Kaili Wang

**Affiliations:** College of Mechanical and Electrical Engineering, Shandong Intelligent Engineering Laboratory of Agricultural Equipment, Shandong Agricultural University Tai'an China xuangt@sina.com; Nanjing Institute of Agricultural Mechanization, Ministry of Agriculture and Rural Affairs Nanjing China zchu369@163.com; Center for Precision and Automated Agricultural Systems, Department of Biological Systems Engineering, Washington State University Prosser USA

## Abstract

Visible and near infrared (Vis-NIR) hyperspectral imaging was used for fast detection and visualization of soluble solid content (SSC) in ‘Beijing 553’ and ‘Red Banana’ sweet potatoes. Hyperspectral images were acquired from 420 ROIs of each cultivar of sliced sweet potatoes. There were 8 and 10 outliers removed from ‘Beijing 553’ and ‘Red Banana’ sweet potatoes by Monte Carlo partial least squares (MCPLS). The optimal spectral pretreatments were determined to enhance the performance of the prediction model. Successive projections algorithm (SPA) and competitive adaptive reweighted sampling (CARS) were employed to select characteristic wavelengths. SSC prediction models were developed using partial least squares regression (PLSR), support vector regression (SVR) and multivariate linear regression (MLR). The more effective prediction performances emerged from the SPA–SVR model with *R*_p_^2^ of 0.8581, RMSEP of 0.2951 and RPD_p_ of 2.56 for ‘Beijing 553’ sweet potato, and the CARS–MLR model with *R*_p_^2^ of 0.8153, RMSEP of 0.2744 and RPD_p_ of 2.09 for ‘Red Banana’ sweet potato. Spatial distribution maps of SSC were obtained in a pixel-wise manner using SPA–SVR and CARS–MLR models for quantifying the SSC level in a simple way. The overall results illustrated that Vis-NIR hyperspectral imaging was a powerful tool for spatial prediction of SSC in sweet potatoes.

## Abstract 2

Visible and near infrared (Vis-NIR) hyperspectral imaging was used for fast detection and visualization of soluble solid content (SSC) in ‘Beijing 553’ and ‘Red Banana’ sweet potatoes.

## Graphical abstract

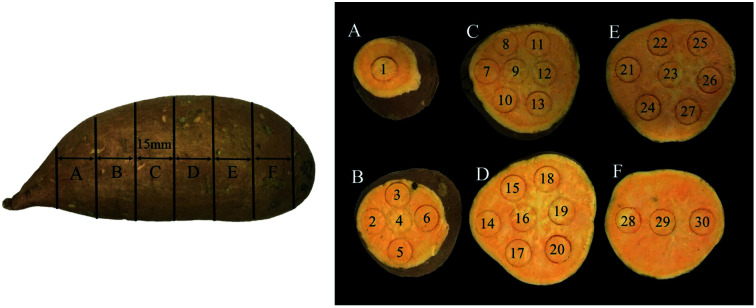

## 1 Introduction

Sweet potato (*Ipomoea batatas* L.) is grown worldwide as a strong adaptive crop to drought, temperature and low fertile soils. It contains plenty of starch, multiple vitamins, protein and inorganic salts such as calcium, phosphorus and iron. Sweet potato has been widely consumed for its functions of delaying aging, improving immunity and preventing cancer.^1,2^ Soluble solids mainly contain sugars, acids, vitamins and minerals,^3,4^ which are the important indicators to determine the taste of sweet potato. Consumers generally prefer sweet potato with high and uniform soluble solid content (SSC). However, SSC distribution in sweet potato varies greatly depending on growth environment such as temperature, moisture and light, which leads to the non-uniform spatial quality.^5^ Whether fresh sweet potatoes or dried chips or slices, SSC plays an important role in their quality attribution and commercial value. Therefore, a strong emphasis should be placed on visual detection of SSC distribution in sweet potato to determine its quality level and develop an online SSC-detection device.

Traditionally, destructive techniques are time-consuming and laborious for measurement of the SSC. Vibrational spectroscopic techniques offer the ability to measure internal quality attributes of agro-food with the ease of application, the non-destructive nature and rapidity.^6,7^ It can impart the inherent chemical and physical information through interactions between electromagnetic radiation and vibrational modes of covalently bound molecules.^8^ Conventional near-infrared (NIR) spectroscopy has been widely employed to predict the gross SSC of diverse fruits such as pear,^9,10^ apples,^11–13^ watermelons,^14^ citrus,^15^ tomatoes,^16^ and sweet cherries,^17^ but was incapable of determining the change in SSC of different positions.

Hyperspectral imaging is a promising technique to obtain spatially resolved spectral information from a sample for present chemical mapping by advances in digital imaging and optics.^18^ As an rapid and non-contact tool, hyperspectral imaging has been applied to measure the internal attributes of foodstuff including total volatile basic nitrogen (TVB-N) content in chicken,^19^ vitamin C content in head cabbage,^20^ as well as SSC and other quality parameters of various fruits.^21–25^ In particular, a partial least squares regression (PLSR) model was developed to map the SSC on apple slices by visible/near-infrared (Vis-NIR) hyperspectral imaging.^26^ A multispectral algorithm was proposed to detect and visual the early decay of citrus with fungal infection.^27^ Hyperspectral imaging was applied to visualize the spatial distribution of protein content in peanuts coupled with chemometrics.^28^ The dry matter in potatoes was detected by Vis-NIR hyperspectral system, and a PLSR model was develop to generate the visualization map for dry matter, with a determination coefficient of prediction set (*R*_p_^2^) of 0.849 and root mean square error of prediction (RMSEP) of 0.878%.^29^ However, to the best of our knowledge, no attention has been paid respect to mapping the distribution of SSC in sweet potato.

The objective of this study was to explore the potential of hyperspectral imaging techniques for spatial prediction of soluble solids content in sweet potato. To this end, hyperspectral images were captured from two cultivars of sliced sweet potatoes, and prediction models were developed for spatial distribution of SSC using preprocessed spectral data and optimal wavelengths.

## 2 Materials and methods

### 2.1 Sample preparation

**Fig. 1:**
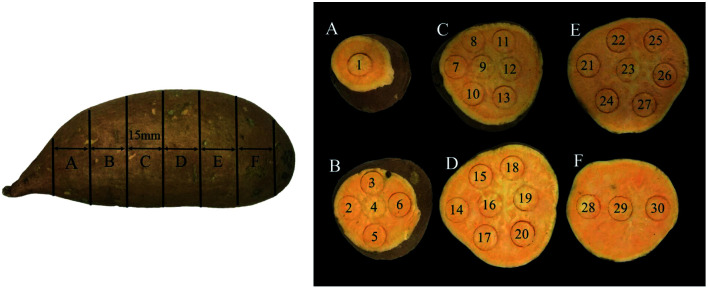
‘Beijing 553’ sweet potato sample.

There were two cultivars of sweet potatoes used for the experiments. ‘Beijing 553’, a yellow-flesh cultivar, and ‘Red Banana’, with orange flesh, were purchased from the XinLv Vegetable Wholesale Market in Tai'an City, Shandong Province, China. A batch of 14 sweet potatoes without any bruises or defects and with uniform shape were selected for each cultivar, and transported to the postharvest engineering laboratory at Shandong Agricultural University, Tai'an City, Shandong Province, China. Then these samples were washed and dried under controlled conditions with 20 °C for 24 h, and weighed. The weight of ‘Beijing 553’ sweet potatoes ranged between 187.39 g and 234.18 g whereas the weight of ‘Red Banana’ sweet potatoes varied between 214.25 g and 267.38 g. Each sweet potato was cut into six slices with 15 mm thick from left to right along its long axis using a slicing tool (ST-100a, Gossoo, China), and residual pieces at both ends were excluded as shown in Fig. 1. Each sliced sweet potato was marked using a puncher with a diameter of 15 mm, and 30 markers were acquired for a single sweet potato, 840 markers in total.

### 2.2 Hyperspectral imaging system

**Fig. 2:**
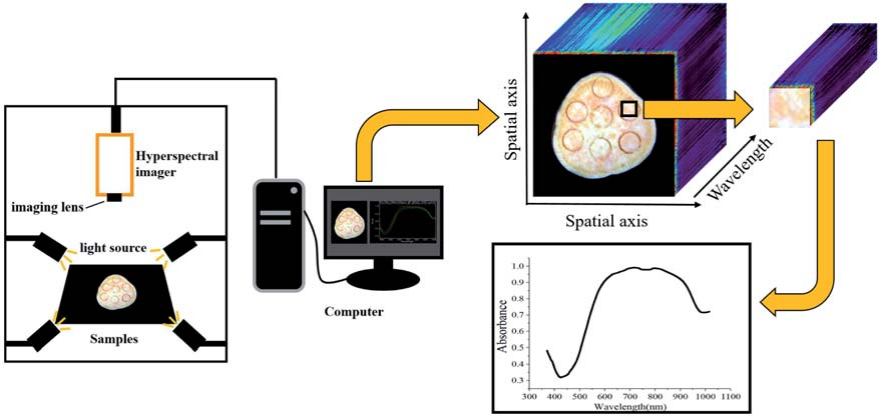
Hyperspectral imaging system.

As shown in Fig. 2, hyperspectral images were captured using a portable hyperspectral imager in 400–1000 nm with 2.8 nm resolution (GaiaField-V10E, Dualix Instruments Co., Ltd, Chengdu, Sichuan Province, China), providing a three-dimensional spectral cube of 1394 (pixels) × 1040 (lines) × 256 (bands). Four 100 W halogen lamps were symmetrically placed around the hyperspectral imager to provide a stable light source with an incident angle of 45°. A special computer was equipped with the hyperspectral data acquisition software SpecView.

### 2.3 Hyperspectral images acquisition and calibration

Before collecting hyperspectral images, the light source was turned on and preheated for 15 minutes to ensure its stability. In order to capture clear and undistorted images, the exposure time was set to 10.38 ms, and the distance between the lens and the sample was 58.72 mm. The white reference image *R*_W_ were captured by scanning the standard white plate with light turned on, and the dark reference image *R*_D_ was obtained through covering the lens without illumination. The raw hyperspectral images *R*_0_ were calibrated as reflectance image *R* to eliminate the impacts of uneven illumination and dark current noise by the following expression.^30^1
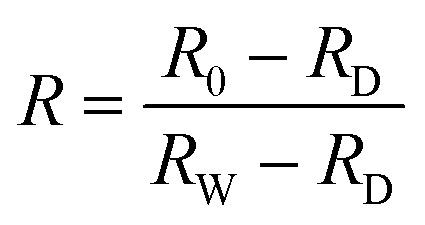


Sliced sweet potatoes were sequentially scanned in the order of slice A to slice F. The region of interest (ROI) (in this case marker) of the corrected images were extracted by ENVI 4.6 (Environment for Visualizing Images software, Research Systems Inc., Boulder, CO, USA). The average spectrum of all the pixels in the ROI was calculated to provide the spectral data for the prediction model.

### 2.4 Measurement of soluble solids content

After hyperspectral images were acquired for each sliced sweet potatoes, a digital refractometer (PAL-1, Atago Co, Tokyo, Japan) was used to measure SSC. Flesh of sweet potato were first scooped out from each marker, and squeezed with manual juicer (B-YZQ001, Bolne, Germany). Then the juice was sucked with a straw and dropped on prism plate of refractometer to show the SSC value on LCD. The measurement was repeated three times to calculate the average value for each marker.

### 2.5 Data processing

#### 2.5.1 Monte Carlo partial least squares

The abnormal samples may seriously affect the performance of prediction models due to errors from instruments and operations. Here, Monte Carlo partial least squares (MCPLS) was used to remove these outliers from the samples using MATLAB 2011a (the Math Works Inc. Natick, MA, USA) and The Unscrambler X 10.4 (CAMO AS, Oslo, Norway) software. Firstly, some fractions of the samples were randomly selected to create the calibration set, and the remaining was assigned to prediction set. Then PLS models were developed multiple times until each sample was used more than once as the prediction set. As a result, each sample obtained a set of predictive residual errors (PRE). Mean value of predicted residual errors (MPRE) and standard deviation of predicted residual errors (STDPRE) were acquired for each sample in the prediction set. Those samples with larger MPRE and STDPRE were identified as abnormal samples.^31^

#### 2.5.2 Sample set partition and spectra pretreatment

Sample set portioning based on joint *x*–*y* distance (SPXY) is an effective sample partition method, which takes into account both spectral characteristics and chemical properties while selecting samples. It has the advantage of improving the predictive ability of the model.^32^ In this study, 840 markers constituted the whole sample set, and were further divided into calibration set and prediction set using MATLAB 2011a software after elimination of abnormal samples.

To greatly improve the prediction ability of SSC in sweet potatoes, spectral data were pretreated to remove noise and other disturbances using baseline correction, de-trending, moving average smoothing (MA), multiplicative scatter correction (MSC), Savitzky–Golay (SG), and standard normal variate (SNV).^33^ A relatively good pretreatment was determined by evaluating the performances of partial least squares regression (PLSR) model.

#### 2.5.3 Characteristic wavelength selection

As a forward variable selection method, successive projection algorithm (SPA) can improve the speed and accuracy of modeling by reducing the collinearity and redundant information between variables. It starts with a certain wavelength and calculates the projection of the wavelength on the unselected wavelength in each iteration. The wavelength with the maximum projection value is selected as characteristic wavelength until the set number of wavelengths reaches. The optimal number of variables is determined by the lowest root mean square error of cross validation (RMSECV) in multiple linear regression (MLR) calibration.^34^ The process of SPA was operated by a graphical user interface GUI_SPA in MATLAB 2011a.

Competitive adaptive reweighting algorithm (CARS) is an effective wavelength variable selection method using the ‘survival of the fittest’ strategy in Darwin's evolution theory. Through *N*-times adaptive reweighted sampling technique, the wavelength variables with large absolute value of regression coefficient are screened out from PLS model, and the wavelength variables with small weight are removed. After *N*-times sampling, *N* subsets of variables are obtained in an iterative manner. Based on 10-fold cross-validation, RMSECV values are calculated for each subset of variables in PLS model, and the subset with the smallest RMSECV is characterized as characteristic wavelengths.^35^ The process of CARS was operated in MATLAB 2011a.

#### 2.5.4 Prediction models

PLSR employs the information from full spectra to predict sample composition. It is used to model the maximum covariance or a linear relationship between reference values (in this case SSC) *Y* and spectral data *X*. In the process of modeling, a smaller amount of new variables in the *X* space were extracted to best describe the *Y* space and reduce the dimensionality.^36,37^

As an extension of support vector machine (SVM), support vector regression (SVR) attempts to cast the original data into a feature space of high dimensionality using nonlinear mapping functions. It conducts the linear relationship between the independent and the dependent variables by adopting the structural risk minimisation principle.^38^ During modeling, three parameters, insensitive loss coefficient *ε*, penalty factor *C*, width coefficient of kernel function (in this case radial basis kernel) *γ*, were optimized using a grid search procedure.

Multiple linear regression (MLR) is a widely used method for modeling the relationship between spectra data and chemical components by the linear equation defined as follows.^39–41^

2 *Y* = *a*_0_ + *a*_1_*X*_1_ + *a*_2_*X*_2_ + … + *a*_*i*_*X*_*i*_ + *ε*

where *Y* denotes SSC value, *a*_*i*_ are the regression coefficient, *X*_*i*_ are the spectral data at different wavelength bands, and *ε* is the regression deviation.

Each model was developed using The Unscrambler X 10.4 and Origin 2017 (Origin Lab Corporation, Northampton, MA, USA) software, the performance of which was evaluated by a determination coefficient of calibration (*R*_c_^2^), determination coefficient of prediction (*R*_p_^2^), root mean square error of calibration (RMSEC), and root mean square error of prediction (RMSEP). Moreover, residual predictive deviation (RPD) was considered for calibration (RPD_c_) and prediction (RPD_p_).

## 3 Results and discussion

### 3.1 Analysis of soluble solids content

**Fig. 3:**
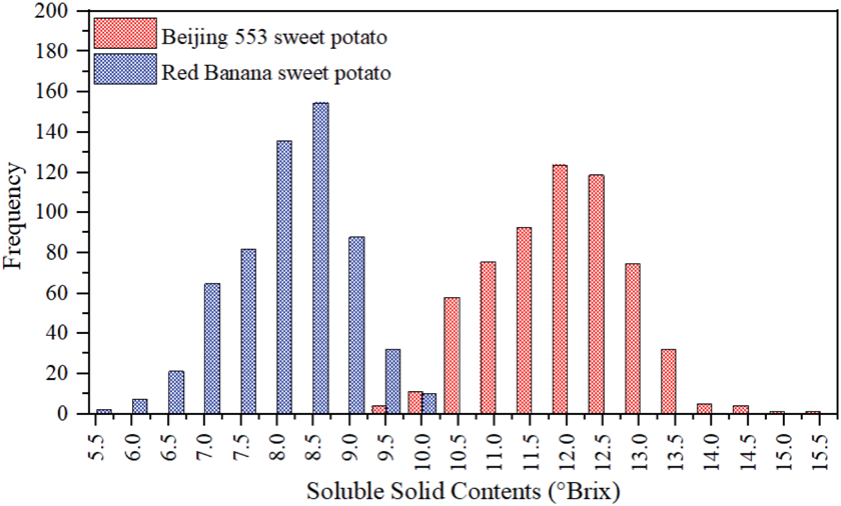
Distribution of SSC in sweet potato.

SSC was measured from 840 ROIs of two cultivars of sweet potatoes. Fig. 3 illustrated the SSC distribution of 840 ROIs. For ‘Beijing 553’ sweet potato, SSC values of 420 ROIs varied from 9.3–15.5 °Brix, and most of them ranged from 10.3–13.0 °Brix. For ‘Red Banana’ sweet potato, SSC values of 420 ROIs varied from 5.5–10.2 °Brix, and most of them ranged from 7.0–9.2 °Brix. It could be seen clearly from the Fig. 3 that SSC values of ‘Beijing 553’ sweet potato were generally higher than that of ‘Red Banana’ sweet potato. Moreover, ‘Beijing 553’ sweet potatoes had a wider SSC distribution than ‘Red Banana’ sweet potato. On the whole, there were notable distinctions in SSC value and its distribution between two cultivars of sweet potatoes.

### 3.2 Spectral curves

**Fig. 4:**
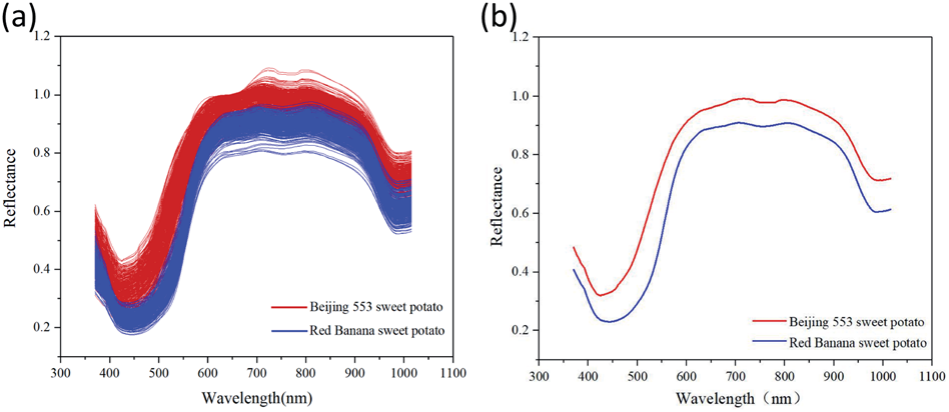
Spectral curves for two cultivars of sweet potatoes. (a) Original spectral curves; (b) mean spectral curves.

Fig. 4 showed the original and mean spectral curves from 420 ROIs for each cultivar of sweet potato. It was found that spectral curves of ‘Beijing 553’ sweet potato were similar to that of ‘Red Banana’ sweet potato, but the former is higher than the latter. The spectral difference in the visible range was caused by the color characteristics of the samples themselves. There was a large absorption peak at around 425 nm, which was the strong absorption band of carotenoids.^29^ The small absorption peak around 650 nm was the strong absorption region of chlorophyll.^42^ Moreover, spectral reflectance had a notable difference in the NIR region as the result of the chemical differences. The peaks at 770 nm and 980 nm were assumed to O–H stretching third- and second-overtone of water respectively, which were relevant to SSC in sweet potatoes.^35^

### 3.3 Elimination of abnormal samples

**Fig. 5:**
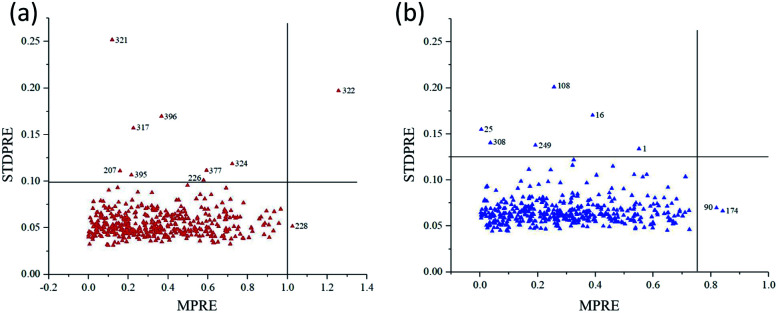
Scatter plot of MPRE and STDPRE for MCPLS. (a) ‘Beijing 553’ sweet potato; (b) ‘Red Banana’ sweet potato.

75% of the samples were randomly selected as the calibration set, and the remaining 25% were used as the prediction set. This process was then repeated 5000 times, and scatter plot of MPRE–STDPRE for MCPLS was illustrated in Fig. 5. For ‘Beijing 553’ sweet potato, there were 10 abnormal samples with MPRE over 1.0 and STDPRE over 0.099. As a result, these abnormal samples were removed including samples 207, 226, 228, 317, 321, 322, 324, 377, 395, and 396 (Fig. 5a). For ‘Red Banana’ sweet potato, there were 8 abnormal samples with MPRE over 0.75 and STDPRE over 0.12. They were samples 1, 16, 25, 90, 108, 174, 249, and 308 (Fig. 5b). The remaining 410 samples of ‘Beijing 553’ sweet potatoes and 412 samples of ‘Red Banana’ sweet potatoes could be used for SSC prediction.

### 3.4 Sample set partition and spectra pretreatment

SPXY algorithm was applied to split the samples into calibration set and prediction set at the ratio of 3 : 1, and the results were shown in Table 1. It could be observed that the maximum and minimum values of SSC occurred in calibration set of two cultivars of sweet potato, and SSC values were widely distributed in the prediction set. Thus sample set partition was reasonable.

**Table 1:** Statistics analysis of measured samples of the calibration set and prediction set

Cultivars	Sample sets	Number	Soluble solid content (°Brix)			
			Min	Max	Mean	SD
Beijing 553	Calibration	308	9.9	13.4	11.8	0.7758
	Prediction	102	10	13	11.9	0.7560
Red banana	Calibration	309	6.1	9.3	8.0	0.6669
	Prediction	103	6.5	9.2	8.0	0.5739

Various spectral pretreatments were explored and evaluated their performances of SSC prediction using PLSR models. SG was the most commonly used method to eliminate noise. SNV and MSC could be applied to eliminate the scattering effect caused by the light and particle size. De-trending could eliminate the baseline drift caused by spectral diffuse reflection. Those methods were considered with good spectral preprocessing ability when PLSR model had the higher *R*^2^ and RPD, and the lower RMSE. Table 2 demonstrated the regression statistics achieved on preprocessed data for calibration and prediction of sweet potatoes. For ‘Beijing 553’ sweet potatoes, RMSEC varied between 0.3224 and 0.3309 with *R*_c_^2^ ranging between 0.8160 and 0.8254, and RMSEP varied between 0.3577 and 0.3687 with *R*_p_^2^ ranging between 0.7713 and 0.7853. The better results were emerged in PLSR models with de-trending pre-treatment due to the higher value RPD_c_ of 2.41 and RPD_p_ of 2.11. For ‘Red Banana’ sweet potatoes, PLSR models with original spectra acquired the relatively good performance as the result of the higher RPD_c_ of 2.12 and RPD_p_ of 1.88. Therefore, spectral data preprocessed with de-trending and original spectra could be used for subsequent analysis of ‘Beijing 553’ and ‘Red Banana’ sweet potatoes, respectively.

**Table 2:** PLSR models of sweet potato SSC using different pretreatment methods^a^

Cultivars	Pretreatment	*R*_c_^2^	RMSEC	RPD_c_	*R*_p_^2^	RMSEP	RPD_p_	PCs
Beijing 553	Original	0.8227	0.3248	2.39	0.7713	0.3687	2.05	15
	Baseline	0.8228	0.3248	2.39	0.7793	0.3633	2.08	15
	De-trending	0.8254	0.3224	2.41	0.7853	0.3577	2.11	13
	MA	0.8192	0.3280	2.37	0.7777	0.3648	2.07	15
	MSC	0.8219	0.3256	2.38	0.7798	0.3625	2.09	13
	SG	0.8160	0.3309	2.34	0.7822	0.3612	2.09	15
	SNV	0.8201	0.3271	2.37	0.7782	0.3643	2.08	14
Red banana	Original	0.7754	0.3146	2.12	0.7331	0.3058	1.88	14
	Baseline	0.7724	0.3182	2.10	0.7024	0.3146	1.82	13
	De-trending	0.7465	0.3358	1.99	0.6733	0.3296	1.74	17
	MA	0.7627	0.3326	2.01	0.7065	0.3146	1.82	15
	MSC	0.7514	0.3327	2.00	0.7029	0.3143	1.82	11
	SG	0.7580	0.3338	2.00	0.7066	0.3145	1.82	14
	SNV	0.7661	0.3226	2.07	0.7007	0.3155	1.82	12

a ‘PCs’ means number of principal components.

### 3.5 Characteristic wavelengths selection

Spectral data of 256 bands contained a large amount of redundant, collinear and overlapping information, which deteriorated the performance of the multivariate calibration models. In this study, SPA and CARS were used to select characteristic wavelengths with the smallest collinearity and least redundancy for improving the modeling efficiency. Through SPA method, 18 characteristic wavelengths from ‘Beijing 553’ sweet potato and 35 characteristic wavelengths from ‘Red Banana’ sweet potato were selected, accounting for 7.03% and 13.67% of the total wavelength variables, respectively. Through CARS method, 19 characteristic wavelengths from ‘Beijing 553’ sweet potato and 36 characteristic wavelengths from ‘Red Banana’ sweet potato were selected, accounting for 7.42% and 14.06% of the total wavelength variables, respectively. All characteristic wavelengths were detailed in Table 3.

**Table 3:** Characteristic wavelengths selected by SPA and CARS methods

Cultivars	Selection methods	Number	Characteristic wavelengths (nm)
Beijing 553	SPA	18	401, 404, 409, 411, 418, 423, 433, 437, 454, 467, 530, 562, 607, 715, 836, 909, 944, 978
	CARS	19	404, 442, 479, 498, 501, 515, 518, 552, 555, 577, 580, 604, 607, 632, 644, 670, 746, 946, 949
Red banana	SPA	35	418, 423, 425, 428, 433, 435, 462, 467, 469, 471, 474, 476, 481, 486, 496, 508, 530, 547, 560, 577, 602, 632, 655, 672, 708, 743, 792, 810, 839, 865, 891, 907, 946, 959, 989
	CARS	36	452, 457, 462, 467, 471, 474, 479, 510, 520, 523, 547, 552, 560, 572, 587, 592, 622, 634, 675, 682, 710, 723, 748, 766, 795, 800, 802, 815, 818, 828, 901, 909, 941, 949, 957, 997

### 3.6 Prediction models for soluble solids content

**Fig. 6:**
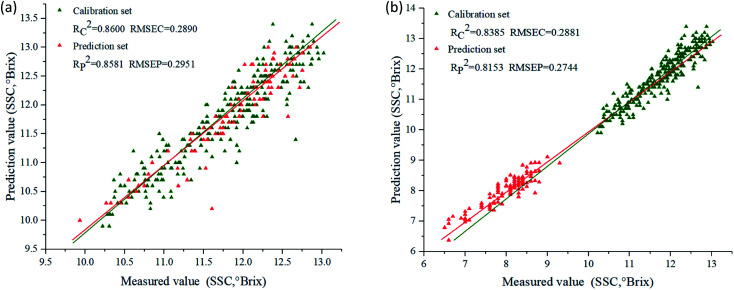
Scatter plots of measured *versus* predicted SSC. (a) SPA–SVR model for ‘Beijing 553’ sweet potato; (b) CARS–MLR model for ‘Red Banana’ sweet potato.

Prediction models for SSC were developed using PLSR, SVR and MLR combined with characteristic wavelengths, and their performances were presented in Table 4. For ‘Beijing 553’ sweet potato, SVR model using characteristic wavelengths selected by SPA (SVR–SPA) provided the best performance with higher *R*_c_^2^ (0.8600) and *R*_p_^2^ (0.8581), lower RMSEC (0.2890) and RMSEP (0.2951). RPD_c_ and RPD_p_ were 2.68 and 2.56, respectively. For ‘Red Banana’ sweet potato, the more effective prediction results were emerged by MLR model using characteristic wavelengths selected by CARS (MLR–CARS), with *R*_c_^2^ of 0.8385 and *R*_p_^2^ of 0.8153, RMSEC of 0.2881 and RMSEP of 0.2744. RPD_c_ and RPD_p_ were 2.31 and 2.09, respectively. Fig. 6 showed the scatter plots of measured *versus* predicted SSC obtained by SPA–SVR and CARS–MLR models, and the predicted SSC values correlated well with measured ones.

**Table 4:** Performance of SSC prediction using PLSR, SVR and MLR models^a^

Cultivar	Model	Spectra	Parameter	Calibration set			Prediction set		
				*R*_c_^2^	RMSEC	RPD_c_	*R*_p_^2^	RMSEP	RPD_p_
Beijing 553	PLSR	SPA	9	0.7318	0.4018	1.93	0.7486	0.3804	1.99
		CARS	10	0.6729	0.4437	1.75	0.6947	0.4191	1.80
	SVR	SPA	(100, 0.1, 0.077)	0.8600	0.2890	2.68	0.8581	0.2951	2.56
		CARS	(100, 0.1, 0.026)	0.8370	0.3057	2.54	0.6909	0.4327	1.75
	MLR	SPA	0.05	0.7318	0.4148	1.88	0.8147	0.3608	2.10
		CARS	0.05	0.6957	0.4426	1.75	0.7969	0.3800	1.99
Red banana	PLSR	SPA	10	0.7361	0.3426	1.95	0.7486	0.3804	1.51
		CARS	13	0.7331	0.3445	1.94	0.6947	0.4191	1.37
	SVR	SPA	(100, 0.1, 0.071)	0.7593	0.3295	2.02	0.7512	0.2875	2.00
		CARS	(100, 0.1, 0.033)	0.8010	0.2686	2.48	0.6728	0.3888	1.48
	MLR	SPA	0.05	0.7518	0.3535	1.89	0.8069	0.3127	1.84
		CARS	0.05	0.8385	0.2881	2.31	0.8153	0.2744	2.09

a Parameter of PLSR model means the optimal number of PCs; parameters of SVR model mean different penalty factor (*C*), insensitivity loss coefficient (*ε*) and width coefficient of kernel function (*γ*), shown as (*C*, *ε*, *γ*); parameter of MLR model means significance level.

### 3.7 Visual distribution of soluble solids content

Spatial distribution maps of SSC in two cultivars of sweet potatoes could be obtained in a pixel-wise manner using SPA–SVR and CARS–MLR models. The specific steps were as follows: (1) obtaining the hyperspectral images of sweet potato slices at characteristic wavelengths; (2) extracting the reflectance of all pixels in the characteristic wavelength image; (3) calculating the SSC corresponding to each pixel point using the prediction models; (4) constructing the spatial distribution maps of SSC in sweet potato slices by pseudo-color processing over gray-scale image.

**Fig. 7:**
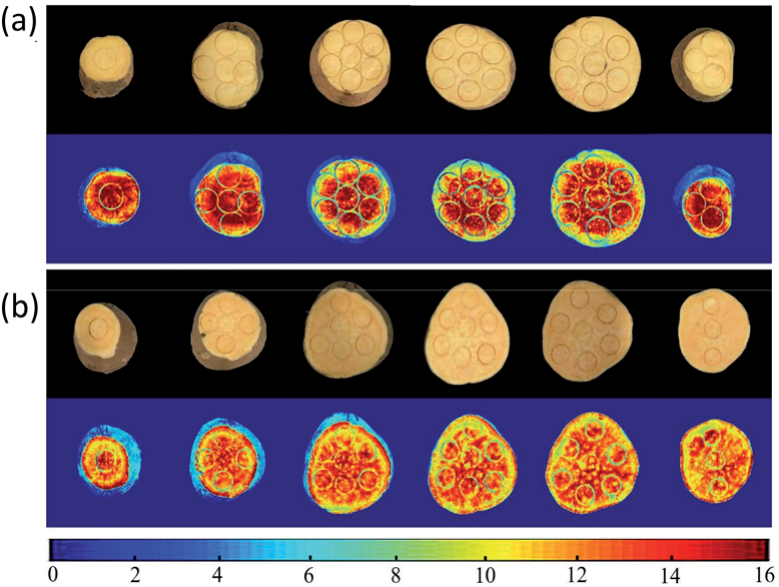
Distribution map of SSC using SPA–SVR model for ‘Beijing 553’ sweet potato (a), and CARS–MLR for ‘Red Banana’ sweet potato (b).

Fig. 7 showed the SSC distribution in sliced sweet potatoes in term of a variation in color from blue to red, higher SSC with intense red color. For ‘Beijing 553’ sweet potato, the 1st, 2nd and 6th slices (from left to right) showed higher SSC values with more red pixels. SSC in other three slices was a significant change from high to low as the result of red at central areas and yellow-green near epidermis. For ‘Red Banana’ sweet potato, central areas and epidermis of six slices have high-SSC red pixels, and other locations showed low-SSC yellow pixels. In general, ‘Beijing 553’ sweet potato had higher SSC than ‘Red Banana’ sweet potato. However, SSC was more uneven within ‘Beijing 553’ sweet potato compared with ‘Red Banana’ sweet potato.

Some studies have been investigated to predict the sugar content in potatoes (another tuber crop) using spectral profiles obtained by hyperspectral imaging, dielectric and nuclear magnetic resonance, and most types of spectral analyses presented a good predictive ability for the average SSC rather than spatial distribution of SSC within potato tubers.^43–46^ In this study, the prediction results acquired from the mapping technique showed the significant differences in SSC occurring spatially within sweet potatoes, which demonstrated hyperspectral imaging as a powerful tool for spatial prediction of SSC in sweet potatoes, laying a foundation for develop an online SSC-detection device.

## 4 Conclusions

This study demonstrated that Vis-NIR hyperspectral imaging was capable of determining the spatial distribution of SSC in sweet potato. SPA–SVR model had the best performance for ‘Beijing 553’ sweet potato with higher *R*_c_^2^ (0.8600) and *R*_p_^2^ (0.8581), lower RMSEC (0.2890) and RMSEP (0.2951). RPD_c_ and RPD_p_ were 2.68 and 2.56, respectively. MLR–CARS model was the more effective for ‘Red Banana’ sweet potato, with *R*_c_^2^ of 0.8385 and *R*_p_^2^ of 0.8153, RMSEC of 0.2881 and RMSEP of 0.2744. RPD_c_ and RPD_p_ were 2.31 and 2.09, respectively. Distribution of SSC in sweet potatoes was visualized and mapped to allow quantifying the SSC level in a spatial way, showing the great potential for quality monitor of sweet potatoes.

## Conflicts of interest

The authors declare that there is no conflict of interests regarding the publication of this study.

## Supplementary Material
